# Finding recurrent RNA structural networks with fast maximal common subgraphs of edge-colored graphs

**DOI:** 10.1371/journal.pcbi.1008990

**Published:** 2021-05-28

**Authors:** Antoine Soulé, Vladimir Reinharz, Roman Sarrazin-Gendron, Alain Denise, Jérôme Waldispühl

**Affiliations:** 1 School of Computer Science, McGill University, Montréal, Canada; 2 LiX, École Polytechnique, Paris, France; 3 Department of Computer Science, Université du Québec à Montréal, Montréal, Canada; 4 Laboratoire de recherche en informatique, Université Paris-Saclay - CNRS, Orsay, France; 5 Institute for Integrative Biology of the Cell (I2BC), Université Paris-Saclay - CEA - CNRS, Gif-sur-Yvette, France; New York University, UNITED STATES

## Abstract

RNA tertiary structure is crucial to its many non-coding molecular functions. RNA architecture is shaped by its secondary structure composed of stems, stacked canonical base pairs, enclosing loops. While stems are precisely captured by free-energy models, loops composed of non-canonical base pairs are not. Nor are distant interactions linking together those secondary structure elements (SSEs). Databases of conserved 3D geometries (a.k.a. modules) not captured by energetic models are leveraged for structure prediction and design, but the computational complexity has limited their study to local elements, loops. Representing the RNA structure as a graph has recently allowed to expend this work to pairs of SSEs, uncovering a hierarchical organization of these 3D modules, at great computational cost. Systematically capturing recurrent patterns on a large scale is a main challenge in the study of RNA structures. In this paper, we present an efficient algorithm to compute maximal isomorphisms in edge colored graphs. We extend this algorithm to a framework well suited to identify RNA modules, and fast enough to considerably generalize previous approaches. To exhibit the versatility of our framework, we first reproduce results identifying all common modules spanning more than 2 SSEs, in a few hours instead of weeks. The efficiency of our new algorithm is demonstrated by computing the maximal modules between any pair of entire RNA in the non-redundant corpus of known RNA 3D structures. We observe that the biggest modules our method uncovers compose large shared sub-structure spanning hundreds of nucleotides and base pairs between the ribosomes of *Thermus thermophilus*, *Escherichia Coli*, and *Pseudomonas aeruginosa*.

## Author summary

Ribonucleic Acids (RNAs) are performing a broad range of essential molecular functions in cells, many of which rely on intricate folding properties of the molecule. Watson-Crick and Wobble base pairs form early, stack onto each other to create stems connected by loops, which are themselves stabilized by more sophisticated base interaction patterns. These networks are essential to shape RNA 3D structures but unfortunately still poorly understood. Here, we undertake the task to build a catalog of base interaction networks occurring in multiple structures. However, a pairwise comparison of all RNA structures is computationally heavy. Therefore, we devise an algorithm leveraging intrinsic properties of RNA base interaction networks that enables us to quickly mine full databases of 3D structures. Compared to previous methods, our techniques bring the total running time of the analysis from months to hours while performing more general searches. The data collected though this work will benefit molecular evolution studies and serve in structure prediction tools.

This is a *PLOS Computational Biology* Methods paper.

## 1 Introduction

Functional RNA tertiary structures are stabilized by a collection of base pairs and base stackings often referred to as the secondary structure. The latter forms a planar structure made of stems of canonical base pairs (i.e. Watson-Crick and Wobble) connected by loops. Although these loops do not feature regular canonical base pairs patterns, they are often characterized by complex non-canonical base pair networks that create sophisticated 3D motifs used to shape the molecular structure. Furthermore, these loops occasionally interact with distant parts of the structure (i.e. other loops or stems) to form bridges stabilizing the global architecture of the RNA. The identification and characterization of these structural sub-units is therefore essential for a better understanding of the evolution of structured RNAs and the development of robust methods for predicting tertiary structures.

**Fig 1:**
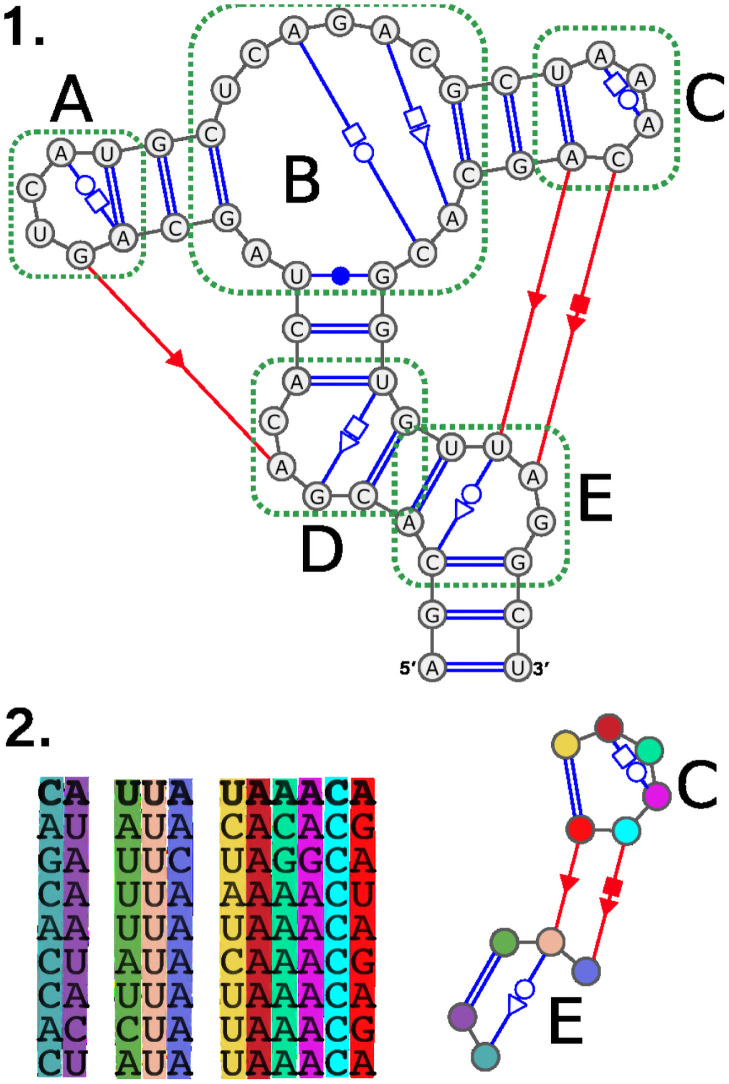
Secondary structure and module. In (1) we show an RNA and its secondary structure with non-canonical interactions. Base pair interactions in blue are local (both nucleotides involved are in the same or in adjacent SSEs) while the ones in red are long range interactions (between two distant SSEs). The canonical base pair interactions are represented with double lines. We highlighted the loops in the structure with green dotted lines. Loops **A** and **C** are hairpins, loops **D** and **E** are interior loops, and loop **B** is a multi-loop. In (2) we show an instance of a module found in the RNA secondary structure in (1). On the right is the base pair pattern that characterizes this module and on the left is the sequence profile of this module (i.e. the nucleotide sequences of the corresponding parts of RNAs this module has been observed in). The first sequence in the profile, for instance, corresponds to the RNA displayed in (1).

RNA modules are small and (usually) densely connected base pair patterns that can be observed in a variety of different molecules, sometimes in multiple locations. Fig 1 displays an RNA secondary structure and, below, a module from the same structure to serve as an illustration. The conservation of RNA modules suggests an evolutionary pressure to preserve specific interaction patterns that constrains the possible set of sequences to the ones compatible with those interactions. As a consequence, identified RNA modules associate sequences to potential structures and so help to draw information about base pairs out of RNA sequences. This information can then be used to infer the 3D structure of the whole molecule [1–7].

Other applications require a well defined and rigorous description of modules. In synthetic biology, the availability of databases of autonomous structural modules is key for designing new molecules [8]. The assembly of RNA binding sites may also require bringing together distant modules within the secondary structure [9]. A comprehensive and indexed catalog of sub-structures would greatly facilitate studies of these sites.

Some RNA modules have received a specific attention such as *GNRA loops*, *Kink-turns*, *G-bulges*, and the various types of *A-minor*s. Moreover, several works have been presented, proposing computational methods to detect RNA modules in tertiary structures using either geometry or graph-based approaches [10–22]. A coarse grain graph representation of the secondary structure with its pseudoknots has already shown the modularity of ribosomal structures [23], and has been used recently for fragment based design applications [24]. However, the purpose of the majority of those methods is to search for known modules in new structures. A couple of methods has been proposed that search local modules without any prior knowledge of their geometry or topology [11, 15]. In addition to those methods, databases of RNA modules found in experimentally determined RNA tertiary structures have been proposed such as RNA 3D Motif Atlas [5] and RNA Bricks [25].

We are interested in the whole landscape of RNA modules (known or not) rather than any RNA module in particular which distinguishes us from most of the works previously mentioned. Furthermore, we aim at extracting recurrent patterns in the secondary structure rather than in the sequence or in the tertiary structure. Those patterns capture a topological information that has been associated with similar tertiary structures and can be in turn used to derive consensus sequences. As previously highlighted in several key structural studies [26, 27], they are therefore interesting RNA modules candidates that warrant further and more quantitative investigations. Our goal is to automatically and comprehensively capture this topological information to accelerate research in area.

To our knowledge, the only published method similar in those aspects is CaRNAval [28], one of our previous work. In CaRNAval, we presented an algorithm to find all identical *interaction networks* between two RNAs [28], which capture the topological information of interaction modules (i.e. RNA modules over two, non-adjacent, secondary structure elements or SSEs) but not the sequences. We made the results of CaRNAval available as an extensive organized catalogue of the *Recurrent Interaction Networks* (RINs) computed on all the non-redundant structures available in RNA3DHub [29]. The method developed for CaRNAval is automated and does not use any prior knowledge of neither the topology nor the geometry of the structures it detects.

CaRNAval was limited in its capacity to capture the whole landscape of RNA modules. The algorithmic work presented in this paper aims to remedy to this. Indeed, by approaching RNA secondary structures as graphs equipped with a *proper edge coloring*, we designed several graph matching algorithms and used them as the core of a modular automated pipeline. Leveraging the *proper edge coloring* of a structure graph allows to improve execution time a hundredfold compared to CaRNAval. Moreover, and this is the main novelty of this method, there are no built-in constraints on the structures it can capture (albeit it accepts such constraints as an optional input). This flexibility joined with the improved performances allow to mine for any kind of RNA module candidates.

Typically, our method can capture structures spanning an arbitrary large number of SSEs when all other approaches are only considering similarities between a loop and CaRNAval only extended this analysis to pairs of loops connected together. We can thus compute similarities between arbitrarily large RNAs. Moreover, we show that the new structures found by removing this restriction complement the landscape of modules presented in CaRNAval and so are other new structures obtained by broadening the search space further. As a consequence, our results underline the universality and fundamental nature of these recurrent architectures.

## 2 Methods

From a set of *mmCIF* files describing 3D structures of RNA chains, we first annotate the interactions with FR3D. The method presented analyze these annotations in four steps.

1. We first build for each chain a directed edge-labelled graph such that the edges represent the phosphodiester bonds as well as the canonical and non-canonical interactions. The labels on the edges correspond to the interaction types plus the indication of the interaction being either local (inside a single SSE) or long-range (between two SSEs)
2. For each pair of RNA graphs, we extract all the Maximal Common Subgraphs such that edges are matched to edges with the same labels
3. Each Maximal Common Subgraph is then processed to obtain the Recurrent Structural Elements (constrained common subgraphs) it contains
4. Finally we gather the Recurrent Structural Elements found together into a non-redundant collection and create a network of direct inclusions.

### 2.1 RNA 2D structure graphs

We rely on RNA 2D structure graphs to represent the structures of RNA chains. RNA 2D structure graphs are directed edge-labelled graphs. Each node represents a nucleotide, each edge represents an interaction (base pair or backbone). Edges are labelled according to the annotation of the interaction they correspond to. Annotations of base pair interactions follow the Leontis-Westhof geometric classification [30]. They are any combination of the orientation cis (c) (resp. trans (t)) with the name of the side which interacts for each of the two nucleotides: Watson-Crick (W, represented with ● in cis orientation or ○ in trans), Hoogsteen (H, ■ in cis □ in trans) or Sugar-Edge (S, ▶ in cis ▷ in trans). Thus, each base pair is annotated by a string from the set: {c,t} × {W,S,H}^2^ or by combining the corresponding symbols. Note that canonical cWW interactions constitute an exception and are represented with a double line instead of “● ●”. Moreover, each basepairs interaction can also be annotated as either *local* or *long range*, depending on the secondary structure elements the nucleotides involved are found in (our method to generate the secondary structure is described in section 3.1). The backbone is represented with directed edges, labelled *b*53.

As a consequence, an annotation (and thus an edge label) is composed of three characters *xYZ* ∈ [*c* ∣ *t*][*W* ∣ *S* ∣ *H*]^2^ plus a parameter *C* ∈ [local ∣ long- range]. Interactions are either symmetric (*xYY*) or not symmetric (*xYZ*). Each non symmetric interaction between nucleobases *xYZ* is complemented by an interaction *xZY* between the same nucleobases and the same value of *C* but in the opposite direction. We introduce an abstract type/label *b*35 to complement the *b*53 label. We can thus define a bijection *ι* as follow:

- *ι*(*xYZ*, *C*) = *xZY*, *C*
- *ι*(*xYY*, *C*) = *xYY*, *C*
- *ι*(*b*53, local) = *b*35, local
- *ι*(*b*35, local) = *b*53, local

An interaction of type *t* between nucleotides *a*,*b* (represented by nodes *v*_*a*_,*v*_*b*_), is represented by two directed edges {*v*_*a*_, *v*_*b*_} and {*v*_*b*_, *v*_*a*_} whose respective labels are *t* and *ι*(*t*). This property is important as a requirement of the algorithms we designed (cf. Section 5.1 in S1 Text).

**Fig 2:**
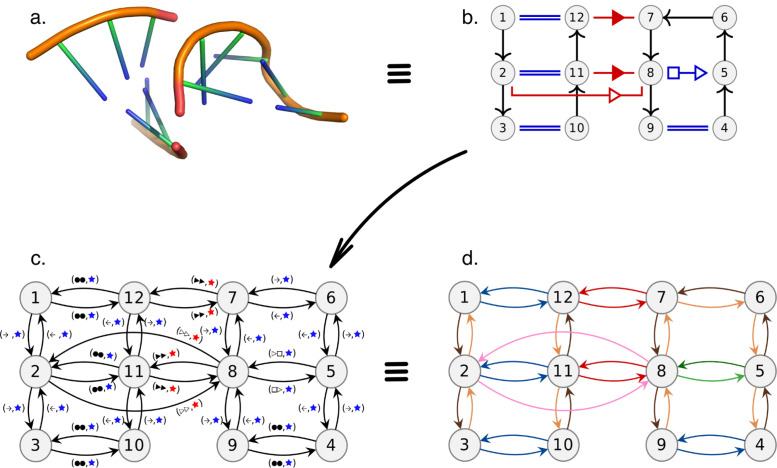
From 3D structure to directed edge-labelled graph. In this figure we illustrate the transition from the 3D structure (a) to RNA 2D structure graph (b) and finally directed edge-labelled graph (c) with a simple RNA structure. Each edge label of the directed edge-labelled graph is a pair which first element represents the type of interaction (using the same symbols as in the RNA 2D structure graph) while the second denotes the local (blue) vs. long-range (red) property of the interaction (using the same colors as in the RNA 2D structure graph). Moreover, the set of edge labels forms a directed *proper edge-coloring*, as illustrated with the last panel (d) where each different geometric type of interaction has been associated a color. Note that panel (d) is only an illustration of the edge labels forming a *proper edge-coloring* as our method does not actually replace the labels by colors.

We represent each RNA chain in the dataset as a RNA 2D structure graph, the annotations of the RNA base pair interactions corresponding to the labels of the edges of the graph (cf. Fig 2).

### 2.2 Graph matching & proper edge-coloring

As we transpose RNA structures into edge-labelled graphs, finding common substructures in the RNA structures comes down to finding common subgraphs in the RNA 2D structure graphs.

Problems that consist in matching graphs or parts of graphs are called *Graph Matching* problems. We are especially interested in finding common subgraphs, an NP-hard problem in general. However, RNA 2D structure graphs inherit some of the constraints of the RNA structures they represent, constraints that translate into a graph property useful for graph matching.

The chemical constraints of nucleotides interactions are such that each edge of a nucleotide should be involved in at most one interaction. This translates in terms of graphs as follows: for all RNA 2D structure graphs *G* = {*V*, *E*} and for all a node *v* ∈ *V*, there are no two edges *e*_1_, *e*_2_ ∈ *E* that originate from *v* with the same label. To put it differently, the set of labels on the edges of any RNA 2D structure graphs naturally forms a *Proper Edge-Coloring* (PEC). We designed three graph matching algorithms designed to take advantage of the proper edge-coloring the RNA 2D structure graphs come equipped with.

### 2.3 Exceptions

We observed a few nucleotides annotated with two interactions involving the same Leontis-Westhof edges in some RNA structures (0.02% of the nucleotides of our reference dataset cf. section 3.1). Those interactions could either be annotation errors or biologically relevant. Given the rarity of those exceptions, we chose to duplicate the graphs concerned into different proper edge-colored versions, each covering a different interpretation. Details about the duplication procedure and the different versions are provided S2 Text.

### 2.4 Graph matching algorithms

In this section we briefly introduce our 3 algorithms, the 3 problems they solve and how we take advantage of the PEC. Extensive and formal descriptions are provided S1 Text.

#### 2.4.1 Definitions & notations

Two graphs *G* = {*V*_*G*_, *E*_*G*_} and *H* = {*V*_*H*_, *E*_*H*_} are isomorphic *iff* there is a bijection *b* from *V*_*G*_ to *V*_*H*_ that respects the edges and their labels. A graph *G* = {*V*_*G*_, *E*_*G*_} is a *subgraph* of graph *H* = {*V*_*H*_, *E*_*H*_} *iff* there exists at least one injection *i* from *V*_*G*_ to *V*_*H*_ that respects the edges and their labels.

Given two graphs *G*,*H*, a graph *S* = (*V*_*S*_, *E*_*S*_) is a *common subgraph* of *G* and *H* if it is a subgraph of *G* and a subgraph of *H*. A common subgraph *S* of *G* and *H* is *maximal* *iff* for all *S’* subgraph of *G* and *H*, *S* ⊂ *S*′ ⇒ *S* = *S*′. All three algorithms take two properly edge-colored graphs *G* = {*V*_*G*_, *E*_*G*_} and *H* = {*V*_*H*_, *E*_*H*_} as an input. For any color *c*, the sets of *c*-colored edges are denoted *E*_*Gc*_ and *E*_*Hc*_.

#### 2.4.2 Using the PEC when extending a matching

**Fig 3:**
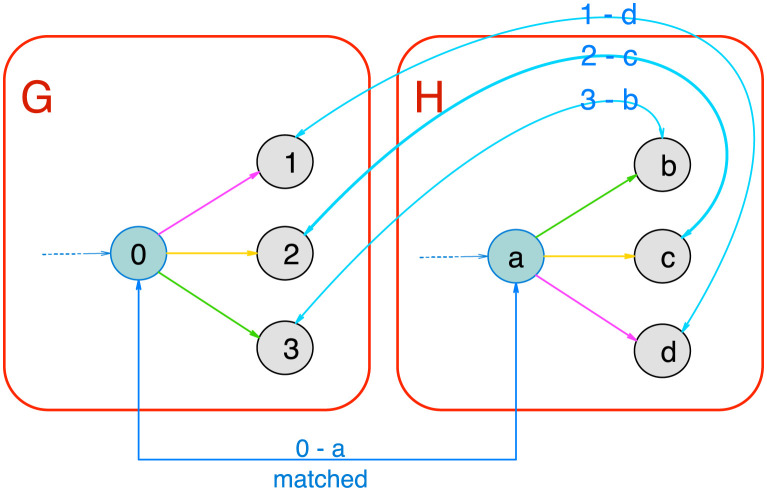
Impact of proper edge-coloring on graph-matching. This figure displays a piece of two graphs (G on the left and H on the right) in which the nodes 0 and *a* are already matched together. The next step is to match their neighbours. In the generic case, all permutations have to be tested. On the contrary, in the example displayed, the colors of the edges limit the options to consider to a single one.

The three algorithms presented in this paper revolve around exploiting the constraint added by having to respect the PEC when matching two graphs to greatly reduce the search space. All three algorithms reliy on the same core strategy. Matching the two graphs is done by starting with a minimal match and then extending it through the neighbors of the already matched nodes. This strategy is common in graph matching and usually requires to test all permutations between the two sets of neighbours. However, the constraint of respecting the PEC only leaves at most a single valid affectation of the neighbours, as illustrated in Fig 3. As a consequence, the complexity of the extension process is linear in the number of nodes (since the number of colors is fixed, cf. S1 Text).

#### Graph isomorphism algorithm

The *Graph Isomorphism* problem consists in determining if two properly edge-colored graphs *G* and *H* are isomorphic. Our Graph Isomorphism Algorithm determines the color *c* that minimizes the product |*E*_*G*,*c*_| × |*E*_*H*,*c*_|. Then, for all pairs of edges ({*g*_1_, *g*_2_}, {*h*_1_, *h*_2_}) ∈ *E*_*G*,*c*_ × *E*_*H*,*c*_, the algorithm launches an extension with the matching ((*g*_1_, *h*_1_), (*g*_2_, *h*_2_)) as starting point. The two graphs are isomorphic *iff* it exists a matching that can be extended into a bijection of *V*_*G*_ and *V*_*H*_ that respects the edges and their coloring. As we mentioned previously, the extension process is in O(|C|×n) (assuming *n* = |*V*_*G*_| = |*V*_*H*_|, if not, *G* and *H* are trivially not isomorphic) and the number of starting point is capped by $O(n^{2}/|C|)$ resulting in a $O(n^{3})$ complexity for the algorithm (cf. S1 Text).

#### 2.4.3 Subgraph isomorphism algorithm

The *Subgraph Isomorphism* problem consists in, given two properly edge-colored graphs *G* and *H*, determining if *G* is a subgraph of *H*. Our Subraph Isomorphism Algorithm is derived from our Graph Isomorphism Algorithm, the difference between the two being that *G* is a subgraph of *H* *iff* it exists a matching that can be extended into an injection of *V*_*G*_ in *V*_*H*_ that respects the edges and their coloring. The complexity is the same as the Graph Isomorphism Algorithm: $O(n^{3})$ with *n* = *min*(|*V*_*G*_|, |*V*_*H*_|) (cf. S1 Text).

#### 2.4.4 All maximal common subgraphs algorithm

**Fig 4:**
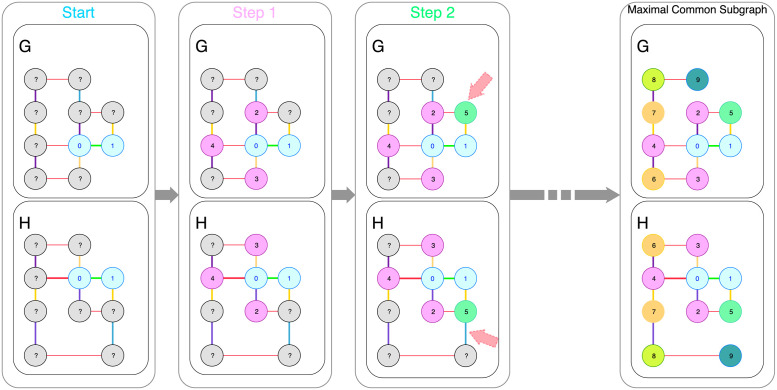
Illustration of the extension process. This figure illustrates the extension process from a “starting point” (here ((*g*_0_, *h*_0_), (*g*_0_, *h*_0_)), in blue). We first consider the neighbors of *g*_0_ and *h*_0_ (in purple). Thanks to the PEC, there is only one way to match them. We then consider the neighbors of *g*_1_ and *h*_1_ (in green). We match *g*_5_ and *h*_5_ but discover that their neighborhoods are not compatible. At this point the behaviours of the three algorithms differ. This discovery implies that the matching cannot be extended to cover all of *G* so the *Graph Isomorphism* and *Subgraph Isomorphism* will abandon it and pass on to another “starting point”. The *All Maximal Common Subgraphs* on the contrary will take note of this discrepancy and keep extending the matching nevertheless. This extension will output a maximal common subgraph of *G* and *H* and a new branch will be created to explore the alternative solution suggested by the discrepancy found.

**Fig 5:**
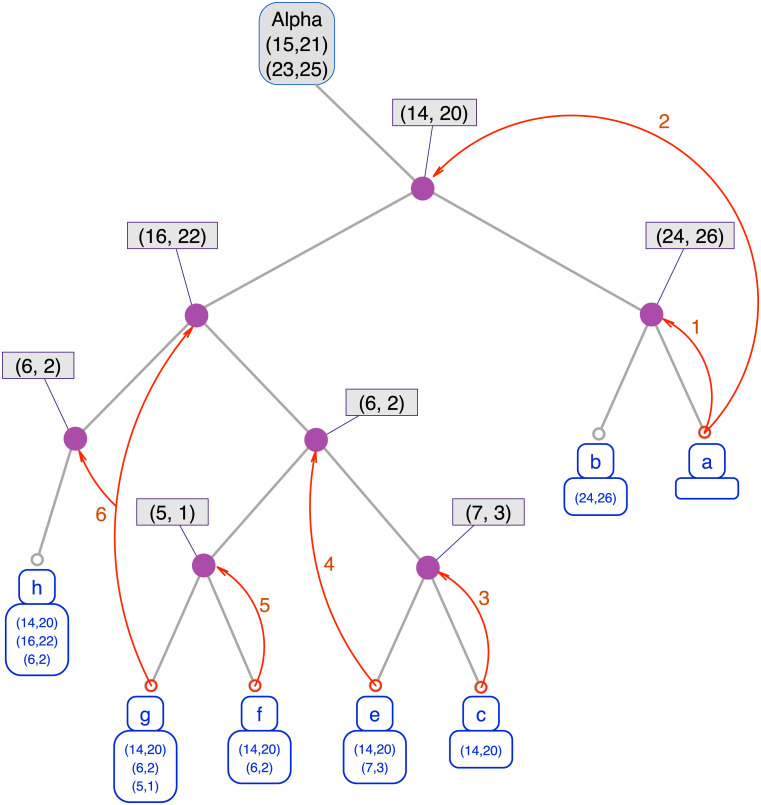
Exploration tree with backtracking. This figure displays the exploration tree representing *a posteriori* the relation between the different branches created. In this tree, the root is a starting point (i.e. the nodes that are already matched at the start of an exploration) and each leaf is a different maximal common subgraph. Each path from the root to a leaf describes an exploration. For instance, the node (14,20) of the exploration tree corresponds to the action of matching the node 14 from G to the node 20 of H. All the leafs in the right subtree have matched 14 to 20 and all the ones in the left subtree have not. Note that only the nodes with a left child are represented, all other nodes have been collapsed since they bear no information about the exploration process. The first exploration always produces the right most maximal common subgraph. In this exemple, the first exploration encountered two conflicts and the algorithm thus produced two new branches which respectively were instructed not to add (24,26) and not to add (14,20). The first of the two produced another maximal common subgraph without any trouble but the second encountered another conflict and so on and so forth.

The *All Maximal Common Subgraphs* problem consists in finding all maximal common subgraphs between two properly edge-colored graphs *G* and *H* (note that this differs slightly from the *maximal common subgraph* problem which usually consists in just finding the largest common subgraph). This algorithm relies on the same extension strategy than the two previous ones. However, unlike the two previous problems, encountering a discrepancy during the extension does not imply that this extension should be abandoned (as illustrated in Fig 4). Instead, it suggests the existence of an alternative way of matching the graphs by considering the nodes in a different order than in the current extension. As we are looking for all maximal common subgraphs, this alternative has to be explored as well. As a consequence, we designed an unconventional backtracking mechanism. For any new discrepancy encountered, we launch a new extension with a list of constraints (similar to instructions) designed to force this new extension to explore the alternative suggested by the discrepancy. Such an extension can also encounter new discrepancies and so on and so forth. Fig 5 illustrates this process and a complete description of this mechanism (with additional illustrations) is provided in S1 Text as well as a formal proof of its correctness.

### 2.5 From common subgraphs back to RNA structures

By transposing the RNA structures to graphs and using our algorithms, we are thus able to obtain the set of *All Maximal Common Subgraphs* contained in any given dataset. However, the number of *Maximal Common Subgraphs* grows exponentially with the size of the dataset and quickly exceeds human capacities. As a consequence, we designed a restriction system to define more human-sized subsets of structural elements and designed our method to extract and organize such subsets specifically rather than the whole set *All Maximal Common Subgraphs*. Those subsets of structural elements are to be defined by users through rules or restrictions, according to the types of structures they want to study.

One of the strong points of our methods is its ability to easily switch from a subset to another since the restriction system is independent from the graph matching part. This opens the opportunity to conduct studies on several related subsets to draw comparisons, as illustrated in section 3. Since we will be working on different subsets simultaneously, let us formalize what those subsets are or can be.

#### 2.5.1 Recurrent Interaction Network (RIN)

We call *Recurrent Interaction Network* (RIN) any recurrent subgraph of RNA 2D Structure Graphs (i.e. observed in at least two RNAs of the dataset). A RIN is formally defined as a pair (S,O) with:

- *S* = {*V*_*S*_, *E*_*S*_} a connected graph with the properties of a RNA 2D structure graph
- O a collection of *occurrences*. An *occurrence* records an observation of *S* in the dataset. We represent an *occurrence* as a pair (*G*, *i*) with *G* = {*V*_*G*_, *E*_*G*_} a RNA 2D structure graph and *i* an injection from *V*_*S*_ to *V*_*G*_ that respects the edge labels.
- $\exists \left(G,i\right),\left(H,i^{\prime }\right)\in O$ s.t. *G* ≠ *H* (i.e. it should be *recurrent*)

This minimal set of properties defines the RIN* class which can be seen as the mother-class from which all other classes are derived by adding additional restrictions. Note that we will be using *class* to refer to subsets of structural elements from now on as the relations between subsets are similar to the ones between the classes of a class-oriented langage.

To illustrate this let us consider a set of additional rules/restrictions *R*, designed to invalidate some structural elements we are not interested in. *R* thus defines RIN^R^ which is a subclass of RIN*. For our method to extract RIN^R^ from a dataset, *R* is to be translated into a filtering function *f*_*R*_: *G* → *C*_*RIN*^*R*^_ with *G* a graph that shares the same properties as an RNA 2D structure graph and *C*_*RIN*^*R*^_ the collection of ${\text{RIN}}_{\text{s}}^{\text{R}}$ in *G* that respects the rules in *R* (the properties defining RIN*are “built-in”). To put simply, the role of *f*_*R*_: *G* → *C*_*RIN*^*R*^_ in the pipeline is to extract the ${\text{RIN}}_{\text{s}}^{\text{R}}$ from the maximal common subgraphs.

Additionally, we offer the possibility of providing a second filtering function $f_{R}^{\prime }:G\to G^{\prime }$ that takes as input an RNA 2D structures graphs *G* in the dataset and outputs another graph *G*′, which is a subgraph of *G* without the edges and nodes in *G* that already infringe a rule of *R* (and thus cannot possibly be part of any valid RIN^R^). $f_{R}^{\prime }$ is optional as it only improves performances by reducing the search space, albeit greatly in most cases.

We will be using RIN^R^ in the following sections to denote an arbitrary class of RINs currently being extracted.

#### 2.5.2 Extraction of RIN^R^

**Fig 6:**
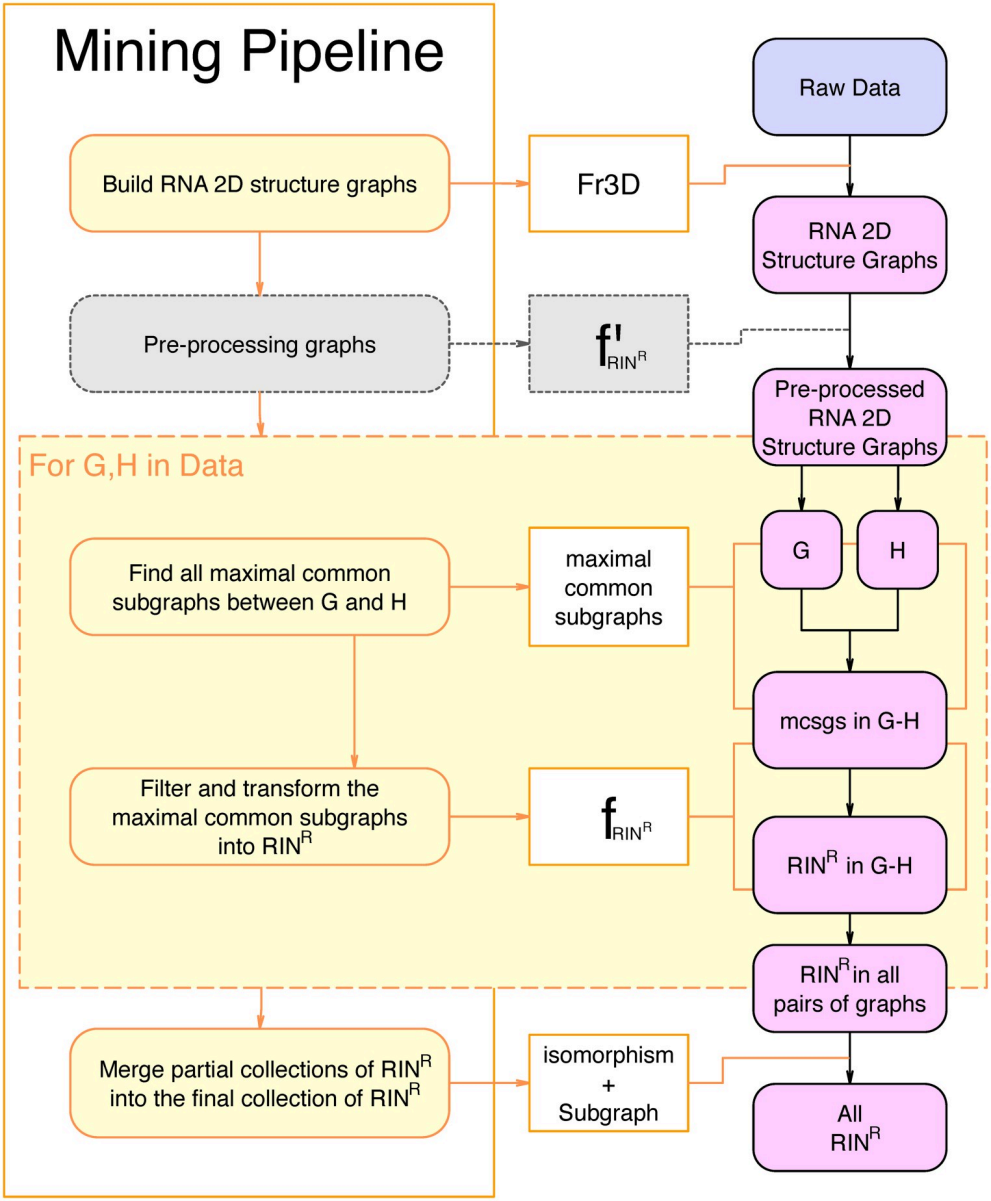
Simplified display of the full pipeline. The RNA 2D structure graphs given as input are pre-processed for the sake of optimization. Each pair of graphs in the pre-processed data is then given to the maximal common subgraphs algorithm as input and the output is post-processed into partial sets of ${\text{RIN}}_{\text{s}}^{\text{R}}$. All partial sets of ${\text{RIN}}_{\text{s}}^{\text{R}}$ are finally merged into the complete set of ${\text{RIN}}_{\text{s}}^{\text{R}}$ which is the output of the whole pipeline.

For every pair of RNA 2D Structure Graphs in the dataset (after the application of $f_{R}^{\prime }$, if provided), we use our algorithm solving the *maximal common subgraphs* problem to extract the set of all maximal common subgraphs between the two graphs (as illustrated in Fig 6). The filtering function *f*_*R*_ (derived from the rules in *R* that defines the class RIN^R^ currently being extracted) is applied to each maximal common subgraph found. The sets of RINs obtained are gathered and clustered using our *graph isomorphism* algorithm. This process involves non trivial but incidental mechanisms which we describe in S2 Text.

Note that our implementation relies on parallelization to improve the performances by distributing the pairs of graphs to process (cf. S2 Text).

#### 2.5.3 Network of RIN^R^

RINs of a given class are often related (i.e. the canonical graph of one may be a subgraph of the canonical graphs of one or several others RINs). In order to display the internal structure of a class of RINs, we organize it into a network *N* = {*V*, *E*}. A node in *V* represents a RIN. An edge *e* = {*r*_1_, *r*_2_} from RIN $r_{1}=\left(S_{1},O_{1}\right)$ to RIN $r_{2}=\left(S_{2},O_{2}\right)$, is in *E* iff *S*_1_ is a subgraph of *S*_2_. If the network is to be displayed, we then remove any edge *e* = {*r*_1_, *r*_3_} ∈ *E* if *e* = {*r*_1_, *r*_2_} ∈ *E* and *e* = {*r*_2_, *r*_3_} ∈ *E* to avoid overloading the display as the edges removed were equivalent to paths in the new version of the network. We rely on our *subgraph isomorphism* algorithm to build those networks efficiently.

## 3 Results & discussion

In this section, we present the results of different applications of our method that fall into two categories. First are the results obtained from the dataset used in CaRNAval [28] that aim at validating our method, at illustrating the flexibility of the method in regards of defining families of substructures and at evaluating the impact of consecutive relaxations of constraints over the same dataset. Second are the results obtained from a recently published dataset that constitute an up-to-date corpus of structures.

### 3.1 Datasets

We use two different datasets of RNA structures. Both datasets are produced from the non-redundant RNA database maintained on RNA3DHub [29]. The difference between the two datasets is the version of this database used: the first dataset is based on version 2.92 (Sept. 9^th^ 2016) whereas the second dataset is based on version 3.137 (Jul. 29^th^ 2020). Our motivations in using two datasets (that will be referred as dataset 2.92 and dataset 3.137 from now on) instead of just using the more recent dataset 3.137 lie in that dataset 2.92 was the one used in CaRNAval [28]. As a consequence, dataset 2.92 was necessary to draw any meaningful comparison with CaRNAval.

The non-redundant RNA database maintained on RNA3DHub [29] contains all-atom molecular complexes with a resolution of at worse 3Å(843 for version 2.92 and 1152 for version 3.137). From these complexes, we retrieved all RNA chains also marked as non-redundant by RNA3DHub (1180 chains for version 2.92 and 1604 for version 3.137). The basepairs were annotated for each chain using FR3D. Because FR3D cannot analyze modified nucleotides or those with missing atoms, our present method does not include them either. If several models exist for a same chain, only the first one was considered.

To distinguish between local and long-range interactions, we define a secondary structure from the ensemble of canonical CWW interactions. This task can be ambiguous for pseudoknotted and large structures. We used the K2N algorithm [31] from the PyCogent library [32]. A case that can not be treated by K2N is when a nucleotide is annotated as having two CWW interactions. Since this is rare, we decided to keep the interaction belonging to the largest stack.

### 3.2 Three different yet related classes of RINs

As we mentioned previously, some of our objectives in this section are to validate our method and evaluate how successive relaxations of rules impact the results. In order to fulfill those objectives, we define three classes of RINs which are successive generalizations obtained by incrementally relaxing rules. Those classes (RIN^abc^, RIN^ab^ and RIN^a^) are named according to the sets of rules they corresponds to so let us first introduce those rules before elaborating on those three classes.

For any RIN={S,O}, where *S* is a *canonical graph* representing the interactions network while O is the collection of occurrences:

*x* - each node in the canonical graph *S* belongs to a cycle in the undirected graph induced by *S* (the undirected graph induced by *S* is obtained by replacing every directed edge by an undirected edge and merging those between the same nodes). We are interested in geometries constrained by annotated interactions.
*y* - if two nodes, *a* and *b* in *S*, form a local canonical base pair, there exists a node *c* in *S* such that *c* is a neighbor to *a* or *b*, and *c* is involved in a long-range or non-canonical interaction. In other words we do not extend stacks which nucleotides are involved in canonical base pairs only. Else, we would match every stem with each other stem.
*z* - each node in *S* is involved in a canonical or a non-canonical interaction (*i.e*. no nodes with only backbone interactions). This impedes chains of nucleotides ony connected by the backbone.
*b* - *S* contains at least 2 long-range interactions, i.e. 4 edges labeled as long-range since each interaction is described with two directed edges. This is a known property of interaction networks joining two SSEs, as the A-minor and the ribose zipper.
*c* - the nucleotides corresponding to the nodes in *S* are captured by exactly 2 SSEs. This was a technical restriction to limit the size of RINs, that could not be handled by the previous algorithms.

Rule *x* aims at enforcing the cohesiveness of the interaction network by preventing danglings that would create variations of little interest. Rule *y* aims at excluding pure stacks of canonical base pairs (i.e. at least two consecutive cWW with no other interaction) which form the core of the structure and are either embedded in the secondary structure with little geometric variation or result from the folding of the tertiary structure (co-axial stacking between helices, loop-loop interactions or pseudo-knots) with often a larger geometric variation. Rules *z* aims at excluding non interacting nucleotides that do not have geometric constraints as interaction networks are intended to capture a representation of the geometry. We will discuss the two last rules in parallel of the description of the classes.

**Fig 7:**
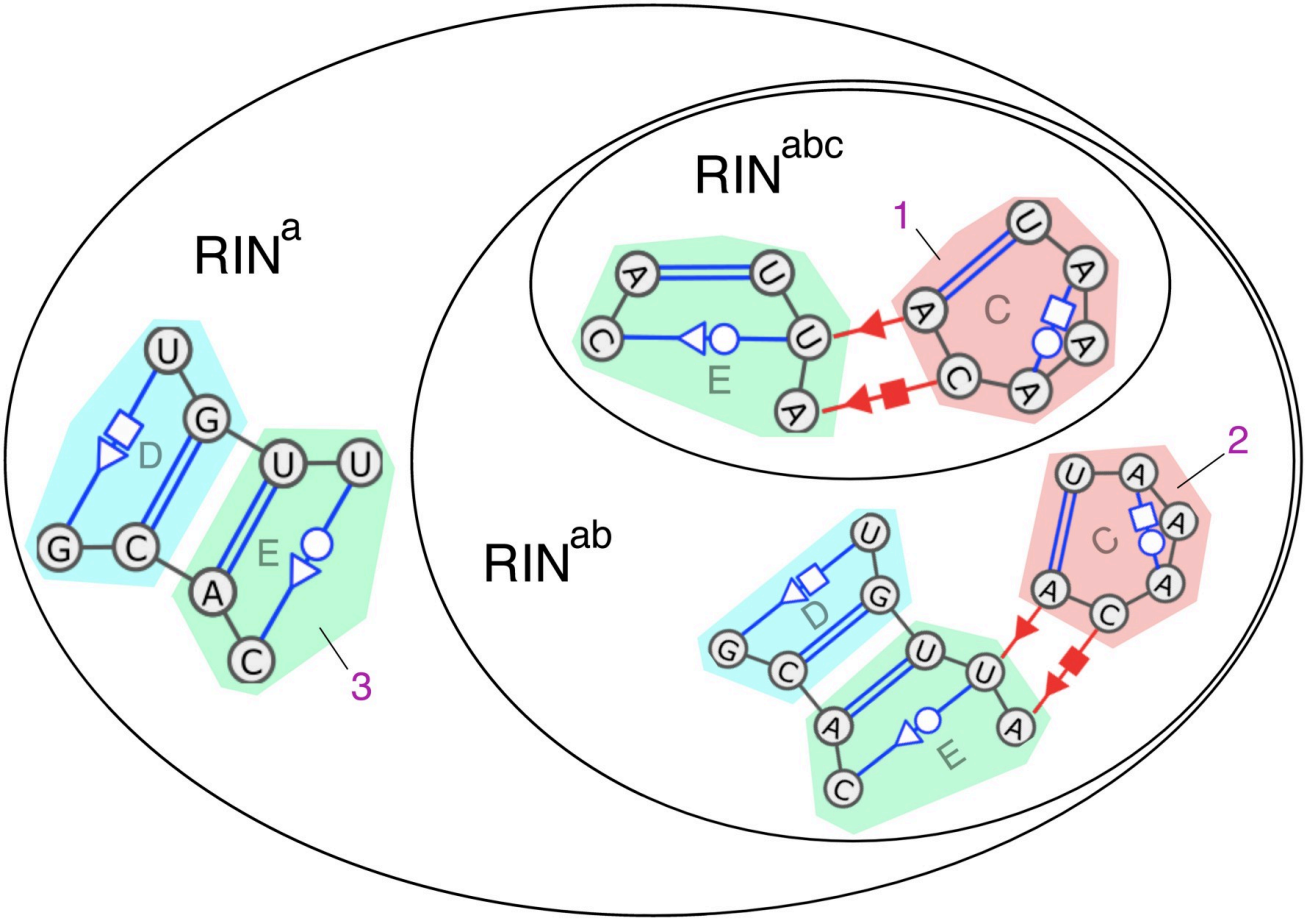
Examples of structures to illustrate the three RIN classes. Those three graphs displayed inside a Venn diagram are subgraphs of Fig 1 with the same SSEs annotations (SSEs D,C and E figured with colored areas). Graph #1 is valid for all three classes. Graph #2 spans over 3 SSEs and so cannot be a valid RIN^abc^. Graph #3 does not contain long-range interactions and thus is only valid for class RIN^a^.

We denote the different RIN classes by concatenating the symbols of the rules that defines them (for instance RIN^xyz^ is the class defined by the first three rules). This naming system has the advantage of making the name of a class an exact description of its definition. However, since the rules *x*,*y*, and *z* are common to all classes, **we will replace xyz with a** in classes’ names. Please refer to Table 1 for a summary of the different classes, their names and the rules they enforces. We also provide examples of structures in Fig 7 to illustrate how the successive relaxations of rules allow additional structures to be captured.

**Table 1:** Rules and RIN classes. Summary of the relation between the rules and the three RIN classes.

Rules↓	Classes→	RIN^abc^	RIN^ab^	RIN^a^
*a*{	Each node is in a cycle	✓	✓	✓
	Stems of canonical base pairs are not extended	✓	✓	✓
	Each node forms at least one base pair	✓	✓	✓
*b*	At least two long range interactions	✓	✓	-
*c*	The entire RIN must be over exactly two SSEs	✓	-	-

We inherit the five rules from the CaRNAval project [28]. The CaRNAval project aimed at extracting RNA structural motifs containing non-canonical base pairs, 2 or more long range interactions and involving exactly 2 SSEs. The set of structures extracted in CaRNAval corresponds in our system to the RIN^abc^ class. We will use the RIN^abc^ class as the reference to validate our method.

We designed the RIN^a^ class to replace the RIN^abc^ as the standard definition of RINs. The RIN^a^ class conserves the core constraints (i.e. *x*,*y* and *z* renamed as *a*) but relaxes secondary constraints *b* and *c*, something that was not possible with the method used in CaRNAval.

We designed the RIN^ab^ class to serve as an intermediary between the RIN^abc^ and RIN^a^ classes. By doing so, we are able to distinguish the impact of relaxing rule *c* from the impact of relaxing rule *b*.

### 3.3 Assessment of the method on Dataset 2.92

#### 3.3.1 Reproduction of previous work

A natural first step in the evaluation of our method is to verify if it is able to reproduce the results presented in CaRNAval. In the notation introduced in the present paper, the collection presented in CaRNAval consists in 331 ${\text{RIN}}_{\text{s}}^{\text{abc}}$ extracted from Dataset 2.92 for a total of 6056 occurrences. Our method extracts those same 331 ${\text{RIN}}_{\text{s}}^{\text{abc}}$ from Dataset 2.92 with the exact same collections of occurrences.

Please note that if CaRNAval extracted 331 ${\text{RIN}}_{\text{s}}^{\text{abc}}$, it displays 337 structures. Indeed, it appears during our evaluation that 4 RINs were actually invalid and should not have have passed the filters of CaRNAval. The absence in our results actually validates our method. The 2 last RINs are a special case: they have only 2 observations with both observations inside a single RNA chain (whereas our definition requires at least two occurrences from distinct RNA chains). As such they are valid RINs but invalid ${\text{RIN}}_{\text{s}}^{\text{abc}}$.

As a conclusion, our method reproduces previous results perfectly as the only discrepancies were due to either errors in said previous results or modifications in definitions.

#### 3.3.2 Relaxing rule *c* →RIN^ab^

Let us now leverage our new method to relax rule *c* and extract ${\text{RIN}}_{\text{s}}^{\text{ab}}$ that are allowed to span over two or more secondary structure elements instead of exactly two for ${\text{RIN}}_{\text{s}}^{\text{abc}}$ (rule *b* still prevents single SSE ${\text{RIN}}_{\text{s}}^{\text{abc}}$).

From Dataset 2.92, we extract 557 ${\text{RIN}}_{\text{s}}^{\text{ab}}$ for a total of 7709 occurrences. Comparing the collection of ${\text{RIN}}_{\text{s}}^{\text{ab}}$ with the collection of ${\text{RIN}}_{\text{s}}^{\text{abc}}$ is not trivial. Indeed, amongst the 557 ${\text{RIN}}_{\text{s}}^{\text{ab}}$, 243 are isomorphic to a RIN^abc^. As a consequence, 88 ${\text{RIN}}_{\text{s}}^{\text{abc}}$ are not matched by a corresponding RIN^ab^. They are instead found inside larger ${\text{RIN}}_{\text{s}}^{\text{ab}}$ (i.e. the canonical graph of the RIN^abc^ is a subgraph of the canonical graph of at least one RIN^ab^), as well as their occurrences. To put it differently, those 88 ${\text{RIN}}_{\text{s}}^{\text{abc}}$ are still captured but are always found inside “larger contexts” that could not be perceived before because of the limitation on the number of SSEs. Now that we relaxed rule *c*, the “larger contexts” are now captured inside ${\text{RIN}}_{\text{s}}^{\text{ab}}$ that “assimilated” those 88 ${\text{RIN}}_{\text{s}}^{\text{abc}}$.

**Fig 8:**
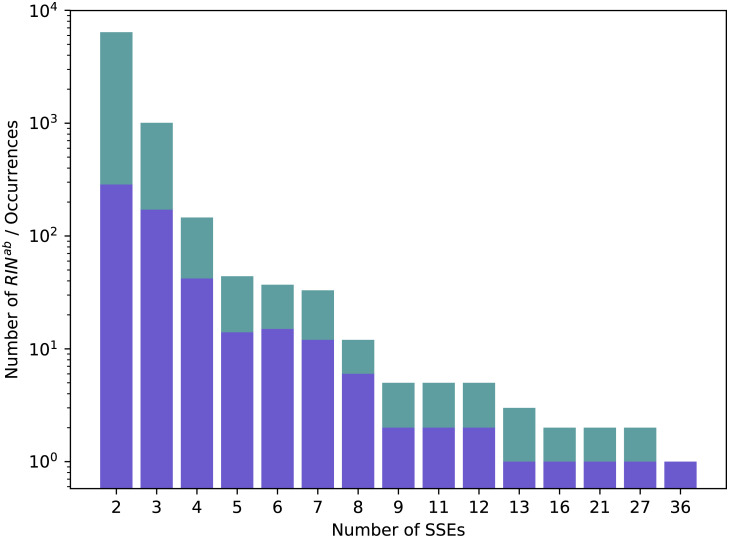
Distribution of ${\text{RIN}}_{\text{s}}^{\text{ab}}$ in Dataset 2.92. Numbers of distinct ${\text{RIN}}_{\text{s}}^{\text{ab}}$(in blue) and all their occurrences (in green) over the different numbers of SSEs they span over in Dataset 2.92.

We show in Fig 8 the distribution of SSEs in the ${\text{RIN}}_{\text{s}}^{\text{ab}}$ and of their occurrences. Please note the logarithmic scale of the *y* axis: relaxing rule *c* indeed allowed larger structures to be extracted but the vast majority of ${\text{RIN}}_{\text{s}}^{\text{ab}}$ span over a small number of SSEs. We will address the very large structures found in separately in section 3.4.2.

Interestingly, the numbers of observations of the 243 ${\text{RIN}}_{\text{s}}^{\text{abc}}/{\text{RIN}}_{\text{s}}^{\text{ab}}$ common to both versions have changed for 81 of them (+4 observations on average). More generally, we observe that relaxing rule *c* also allowed ${\text{RIN}}_{\text{s}}^{\text{ab}}$ to contain varied numbers of SSEs. We show in Table 2 that this variation is nevertheless limited: out of the 557 ${\text{RIN}}_{\text{s}}^{\text{ab}}$, 435 had all of their occurrences span the same number of SSEs. There are 116 that can be over two different number of SSEs, and only 6 ${\text{RIN}}_{\text{s}}^{\text{ab}}$ have their occurrences cover three different number of SSEs.

**Table 2:** ${\text{RIN}}_{\text{s}}^{\text{ab}}$ and variation on SSEs span. For each RIN^ab^ we compute how the number of SSEs covered varies between the occurrences. A value of 0 means that all occurrences are over the same number of SSEs while ±1 (resp. ±2) means that the RIN^ab^ can span two different number of SSEs (resp. three).

Variation in number of SSEs	0	±1	±2
Numbers of ${\text{RIN}}_{\text{s}}^{\text{ab}}$	435	116	6

#### 3.3.3 Relaxing rule *b* →RIN^a^

In the previous section we created the RIN^ab^ class as a generalization of the RIN^abc^ class. A natural way to generalize the problem further is to remove the constraint of having 2 or more long range interactions. We call RIN^a^ the class obtained from RIN^ab^ by removing rule *b* (cf. definition of the classes in 3.2). While this modification is trivial to implement, it does increase the search space drastically compared to the extraction of RIN^ab^. However the collection are way easier to compare.

Indeed, our method finds 920 ${\text{RIN}}_{\text{s}}^{\text{a}}$ for a total of 12239 occurrences and all 557 ${\text{RIN}}_{\text{s}}^{\text{ab}}$ are matched by RIN^a^(and so are their occurrences).

Unlike the relaxation of rule *c* that caused a rearrangement of the collection, relaxing rule *c* does not open the possibility of finding new larger “including” structures. As a consequence, the collection of ${\text{RIN}}_{\text{s}}^{\text{a}}$ is strictly including the collection of ${\text{RIN}}_{\text{s}}^{\text{ab}}$.

**Fig 9:**
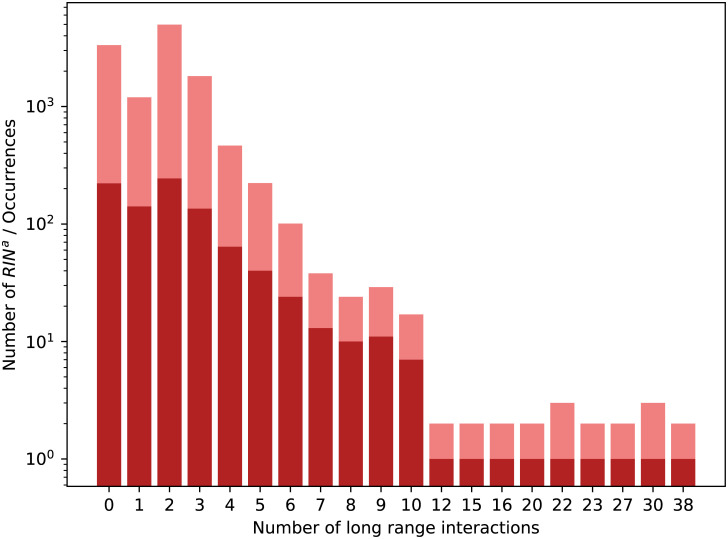
Distribution of ${\text{RIN}}_{\text{s}}^{\text{a}}$ in Dataset 2.92. Numbers of distinct ${\text{RIN}}_{\text{s}}^{\text{a}}$ (in red) and all their occurrences (in rose) over the different numbers of long range interactions they contain in Dataset 2.92.

**Fig 10:**
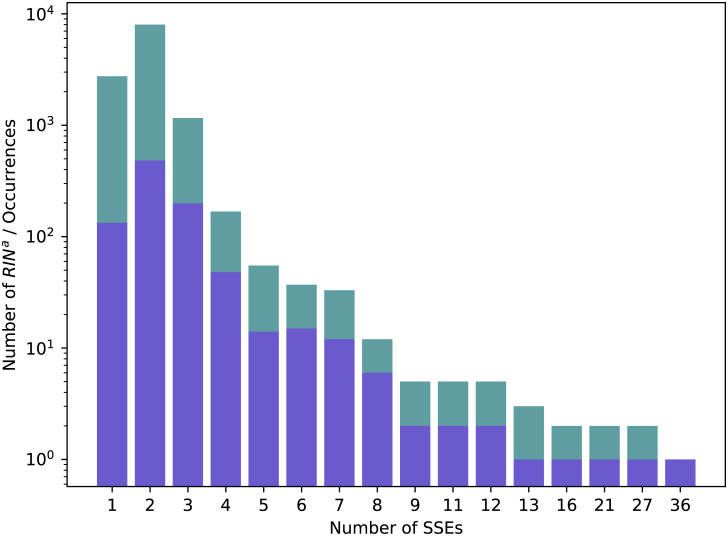
Distribution of ${\text{RIN}}_{\text{s}}^{\text{a}}$. Numbers of distinct ${\text{RIN}}_{\text{s}}^{\text{a}}$ (in blue) and all their occurrences (in green) over the different numbers of SSEs they span over in Dataset 2.92.

The new structures that make the difference between the two collections are ${\text{RIN}}_{\text{s}}^{\text{a}}$ that contain either 0 or 1 long range interaction. We show in Fig 9 the distribution of the ${\text{RIN}}_{\text{s}}^{\text{a}}$ and of their occurrences depending on the number of long range interactions they have. Amongst the new 363 ${\text{RIN}}_{\text{s}}^{\text{a}}$, 222 contain no long range interaction and 141 have exactly 1. Those represent 39% of the ${\text{RIN}}_{\text{s}}^{\text{a}}$ and 37% of the occurrences. In Fig 10 we show the distribution of the number of SSEs the ${\text{RIN}}_{\text{s}}^{\text{a}}$ span over. As expected, the distribution is very similar with its equivalent for ${\text{RIN}}_{\text{s}}^{\text{ab}}$ displayed in Fig 8. The two differences being the additional bar in Fig 10 that corresponds to ${\text{RIN}}_{\text{s}}^{\text{a}}$ that span over exactly one SSE and a higher second bar (i.e. more ${\text{RIN}}_{\text{s}}^{\text{a}}$ spanning over 2 SSEs than ${\text{RIN}}_{\text{s}}^{\text{ab}}$). Similarly to ${\text{RIN}}_{\text{s}}^{\text{ab}}$, the occurrences of a single RIN^a^ span over a consistent number of SSEs as shown in Table 3. Table 4 summarises the numbers of RINs found for each class.

**Table 3:** Variation in the number of SSEs over the occurrences of the same RIN^a^. (Cf. Table 2). Those numbers show that the variation in the number of SSEs amongst the occurrences of a given RIN^a^ is both uncommon and limited, even more than with RIN^ab^, albeit slightly (82% of ${\text{RIN}}_{\text{s}}^{\text{a}}$ with no variation vs 78% of ${\text{RIN}}_{\text{s}}^{\text{ab}}$).

Variation in number of SSEs	0	±1	±2
Numbers of RIN^a^	754	159	7

**Table 4:** Summary of numbers of unique RINs found in the different classes with the total numbers of occurrences. Please note that this table also displays the numbers for the RIN^a^ class in Dataset 3.137 that we will present in section 3.4.

	Dataset 2.92		Dataset 3.137	
Class	unique	occurrences	unique	occurrences
RIN^abc^	331	6056	-	-
RIN^ab^	557	7709	-	-
RIN^a^	920	12239	1875	29344

#### 3.3.4 Networks of RIN^abc^, of RIN^ab^ and of RIN^a^

Let us now compare the collections of RIN^abc^, of RIN^ab^ and of RIN^a^ through the networks they form (cf. section 2.5.3). The network formed by the ${\text{RIN}}_{\text{s}}^{\text{abc}}$ consists in 3 main connected components and named after a characteristic motif they contain. They are the Pseudoknot mesh, the A-minor mesh and the Trans W-C/H mesh, respectively containing 59, 196 and 22 ${\text{RIN}}_{\text{s}}^{\text{abc}}$. The remaining ${\text{RIN}}_{\text{s}}^{\text{abc}}$ are shared between 25 other components of size ranging from 1 to 4.

In contrast, the network of RIN^ab^ only has 16 components compared to the 28 of the RIN^abc^ network. It suggests that the newly found ${\text{RIN}}_{\text{s}}^{\text{ab}}$ connect components of the RIN^abc^ network together. This claim is supported by the fact that, in the network of RIN^ab^, the Pseudoknot and A-minor meshes have merged into a single one containing 482 ${\text{RIN}}_{\text{s}}^{\text{abc}}$. This new giant mesh contains all the elements in the two main meshes presented in CaRNAval plus 230 extra ${\text{RIN}}_{\text{s}}^{\text{ab}}$. The Trans W-C/H mesh remains disconnected and gains 16 elements for a total of 38 RIN^ab^.

The addition of the new structures from the RIN^a^ collection to the RIN^ab^ network connects almost all the nodes of the network. Indeed 888 of the 920 ${\text{RIN}}_{\text{s}}^{\text{a}}$ are inside a single giant component. This component gathers not only the Pseudoknot and the A-minor meshes of the RIN^abc^ network (like the main component of the RIN^ab^ network did), but also the Trans W-C/H mesh. Of the remaining 32 ${\text{RIN}}_{\text{s}}^{\text{a}}$ that are not in this component, 22 are singletons and 10 form 4 different small components. In summary, the RIN^a^ network shows that the RIN^a^ class forms a unified and nearly totally connected landscape of structures.

#### 3.3.5 Performances

We previously mentioned that our method was significantly more efficient than the only published method it can be compared to (i.e. CaRNAval). This statement is to be considered in the context of graph matching and thus NP-hard problems in general. Just like CaRNAval, our method is exponential in the worst case. However our method is able to perform the same task significantly faster than CaRNAval (0.7h instead of 330h). Moreover, our method can extract RIN classes that are beyond the limits of the method of CaRNAval (RIN^a^, RIN^ab^, RNA 3D modules cf. section 3.5).

Table 5 displays several runtimes from our method and the method of CaRNAval on the same machine (20 CPUs) on an indicative basis. In addition to the runtimes on Dataset 2.92, it also displays runtimes on Dataset 3.137 that we will present in the next section 3.4 and on RNA 3D modules that we present in section 3.5. Please note that, for our method, producing the set of ${\text{RIN}}_{\text{s}}^{\text{abc}}$ is equivalent to producing the set of ${\text{RIN}}_{\text{s}}^{\text{ab}}$ and applying a filter corresponding to rule *c*. To put it differently, our method cannot take advantage of rule *c* and so its runtime for class RIN^abc^ is similar to its runtime for class RIN^ab^.

**Table 5:** Runtimes over 20 CPUs. This table displays the runtime of previous method (CaRNAval) and our method (others rows) for different classes of structures extracted. The values have been measured with the linux *time* command and are *real CPU times* i.e. clock time elapsed between the start and the end of the execution. All runs have been performed on the same machine.

	Dataset 2.92	Dataset 3.137
CaRNAval	330h	-
RIN^abc^	0.7h	-
RIN^ab^	0.7h	-
RIN^a^	1.4h	1.8h
RNA 3D modules	-	29h

### 3.4 ${\text{RIN}}_{\text{s}}^{\text{a}}$ from Dataset 3.137

Now that we have accessed our method on Dataset 2.92, let us move to Dataset 3.137. We will only consider RIN^a^ in this section.

As we mentioned when we introduced the two datasets, Dataset 3.137 contains significantly more RNA chains than Dataset 2.92 (1152 vs 843, +37%), a consequence of the four years that separate the publication of the two versions of the non-redundant RNA database maintained on RNA3DHub they are respectively based on.

Our methods finds 1875 distinct ${\text{RIN}}_{\text{s}}^{\text{a}}$ for a total of 29344 occurrences in Dataset 3.137. Compared to the results we obtained from Dataset 2.92 (920 ${\text{RIN}}_{\text{s}}^{\text{a}}$ for a total of 12239 occurrences), we find 104% more ${\text{RIN}}_{\text{s}}^{\text{a}}$ and 140% more occurrences. Those numbers might appear surprising considering that there are only 37% more structures in Dataset 3.137 than in Dataset 2.92. However the structures in Dataset 3.137 are larger in average (65 nt. vs 45nt., +44%) so there are actually nearly twice as much nucleotides in total in Dataset 3.137 than in Dataset 2.9.

Let us mention that all our structures of interest (A-minor type I/II, Ribozipper, GNRA, A-rich Loop) are present amongst those 1875 ${\text{RIN}}_{\text{s}}^{\text{a}}$ and that their numbers of occurrences increased similarly to the average as displayed in Table 6

**Table 6:** Number of occurrences found in Dataset 2.92 and Dataset 3.137 for 5 structures of interest. The 5 structures of interest are denoted using both their name in the litterature (first column) and their ID in our database (second column). Note that it is the same ID displayed in CaRNAval.

RIN^a^	ID	occ. in 2.92	occ. in 3.137	Variation
A-minor Type I	#2	194	411	+111%
A-minor Type II	#17	102	205	+100%
Ribose zipper	#11	133	321	+141%
GNRA	#44	33	71	+115%
A-rich Loop	#74	13	34	+161%

**Fig 11:**
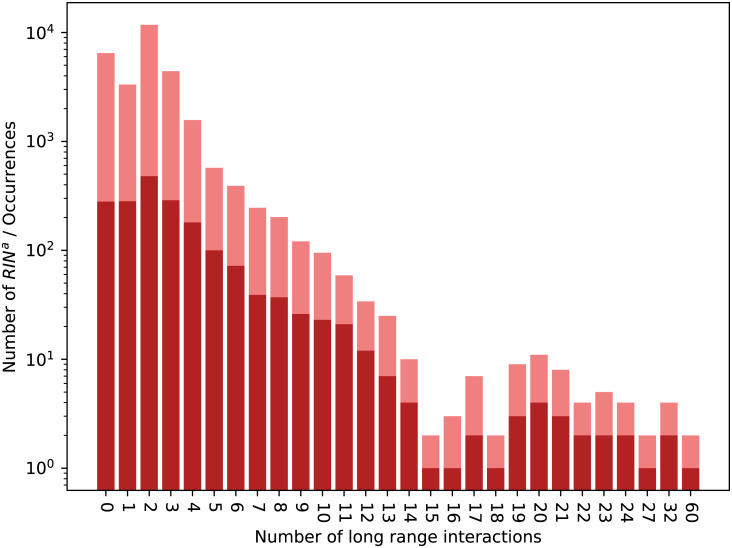
Distribution of ${\text{RIN}}_{\text{s}}^{\text{a}}$ in Dataset 3.137. Numbers of distinct ${\text{RIN}}_{\text{s}}^{\text{a}}$ (in red) and all their occurrences (in rose) over the different numbers of long range interactions they contain in Dataset 3.13.

**Fig 12:**
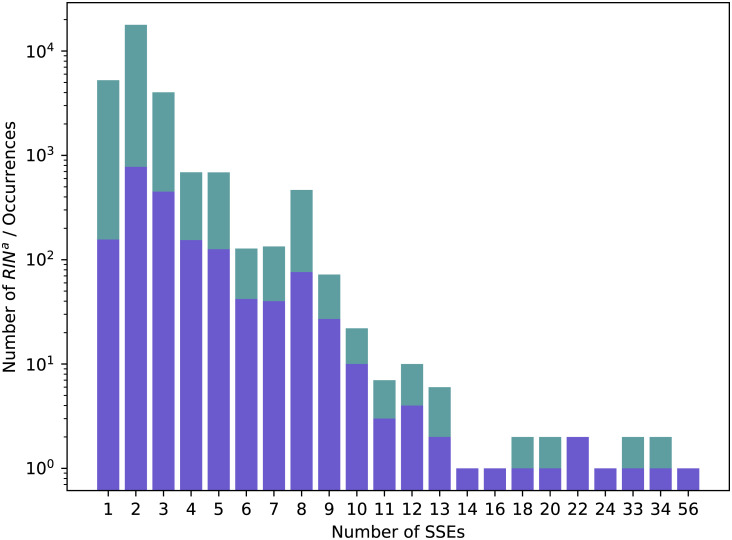
Distribution of ${\text{RIN}}_{\text{s}}^{\text{a}}$ in Dataset 3.137. Numbers of distinct ${\text{RIN}}_{\text{s}}^{\text{a}}$ (in blue) and all their occurrences (in green) over the different numbers of SSEs they span over in Dataset 3.13.

Figs 11 and 12 respectively display the distribution of ${\text{RIN}}_{\text{s}}^{\text{a}}$ by the number of long range interactions they contain and by the number of SSEs they span over. Those distributions are similar to their counterparts from Dataset 2.92 (Figs 9 and 10).

#### 3.4.1 Network of ${\text{RIN}}_{\text{s}}^{\text{a}}$ in Dataset 3.137

The network of ${\text{RIN}}_{\text{s}}^{\text{a}}$ in Dataset 3.137 shows the same trend as in Dataset 2.92. One massive mesh clusters 97.5% of ${\text{RIN}}_{\text{s}}^{\text{a}}$ (vs. 96.5% in Dataset 2.92). This component still aggregates the three meshes (Pseudoknot mesh, A-minor meshe and Trans W-C/H mesh) presented in CaRNAval. The remaining 44 ${\text{RIN}}_{\text{s}}^{\text{a}}$ are distributed in 4 small components (sizes: 7, 3, 3 and 2) and 29 singletons.

#### 3.4.2 Ribosomes and very large ${\text{RIN}}_{\text{s}}^{\text{a}}$

Note: this discussion on the very large ${\text{RIN}}_{\text{s}}^{\text{a}}$ extracted could arguably falls into section 3.3 as it involves the RIN^abc^ and ${\text{RIN}}_{\text{s}}^{\text{ab}}$ classes and thus Dataset 2.92. Yet, some aspects ot this discussion require our latest results on Dataset 3.137 and it was thus moved to section 3.4 instead.

We previously mentioned that the relaxation of rule *c* (*being exactly over 2 SSEs*) allowed for larger structures to be extracted. Indeed, our method does not cap the size of the structures extracted outside of the limitations fixed by the rules. As such, relaxing rule *c*, that directly limits the size of the structures accepted, naturally results in larger structures being found. However, the three rules *x*,*y* and *z* (that we amalgamated into rule *a*) ensure that only densely connected structures are accepted. Typically, if we apply a filter enforcing those rules to the vast majority of the RNA 2D structure graphs in the dataset (which is the role of the second filtering function $f_{{RIN}^{\text{a}}}^{\prime }$ cf. section 2.5.1), it disconnects the vast majority of them as the filter “cuts” the stems, the backbone and the danglings if those do not contain any non-canonical interactions.

Yet, relaxing rule *c* still drastically raised the order (i.e. number of nodes, although the same can be said for the size) of the structures found: while the largest RIN^abc^ found in Dataset 2.92 contained 26 nodes, 64 ${\text{RIN}}_{\text{s}}^{\text{ab}}$ were found with more than 26 nodes on the same dataset. Amongst those 64 ${\text{RIN}}_{\text{s}}^{\text{ab}}$, 4 have more than 100 nodes and the largest contains 293 nodes. The numbers are the same for class RIN^a^ on Dataset 2.92 but not on Dataset 3.137. On Dataset 3.137 there are 287 ${\text{RIN}}_{\text{s}}^{\text{a}}$ over 26 nodes, 7 over 100 and the largest RIN^a^ now contains 376 nodes. By comparison, the number of nodes of our target structures varies between 3 (A-minor Type I) and 13 (A-rich Loop).

Those large ${\text{RIN}}_{\text{s}}^{\text{a}}$ have very limited numbers of occurrences. The 287 ${\text{RIN}}_{\text{s}}^{\text{a}}$ with more than 26 nodes totalize 1154 occurrences for an average of 4 occurrences per large RIN^a^ whereas the average for the whole RIN^a^ class is 15.7 occurrences per RIN^a^. This tendency is even clearer for the largest ones as the 14 largest ${\text{RIN}}_{\text{s}}^{\text{a}}$ only have two occurrences. By comparison, the A-minor Type I and A-rich loop we just mentioned have respectively 411 and 34 occurrences.

A deeper look at those occurrences, and thus the RNA chains those large ${\text{RIN}}_{\text{s}}^{\text{a}}$ are found in, shows that 282 of those 287 large ${\text{RIN}}_{\text{s}}^{\text{a}}$ are found exclusively in ribosomal RNAs (25 RNA chains of various ribosomal subunits from various species). The 5 exceptions are found exclusively in homologues of the SAM-I riboswitch (4 RNA chains) and the largest RIN^a^ of them contains only 40 nodes (128^*th*^ largest RIN^a^).

Large ${\text{RIN}}_{\text{s}}^{\text{a}}$ being nearly only found in ribosomal chains is likely the consequence of ribosomal chains being both significantly larger than the average and heavily structured (which limits the disconnection phenomenon mentioned above). Moreover, both Dataset 2.92 and 3.137 include multiple ribosomal chains despite being non-redundant due to those chains corresponding to different ribosomal subunits and/or organisms.

Those observations on the large ${\text{RIN}}_{\text{s}}^{\text{a}}$ suggest that part of the collection of ${\text{RIN}}_{\text{s}}^{\text{a}}$ (typically the ${\text{RIN}}_{\text{s}}^{\text{a}}$ found in ribosomal chains) could be used as base for a study of conserved structural elements in ribosomes. However, such study falls out of the scope of this paper. On the contrary, and as we mentioned in the previous section, 97.5% of the network of ${\text{RIN}}_{\text{s}}^{\text{a}}$ in Dataset 3.137 is connected in a single component. All those 288 large ${\text{RIN}}_{\text{s}}^{\text{a}}$ are in this giant component and thus are linked to 97.5% of the collection. As a consequence, in the perspective we adopt in this study, those large ${\text{RIN}}_{\text{s}}^{\text{a}}$ constitute the tail-end of our collection of ${\text{RIN}}_{\text{s}}^{\text{a}}$ rather than a separate group.

**Fig 13:**
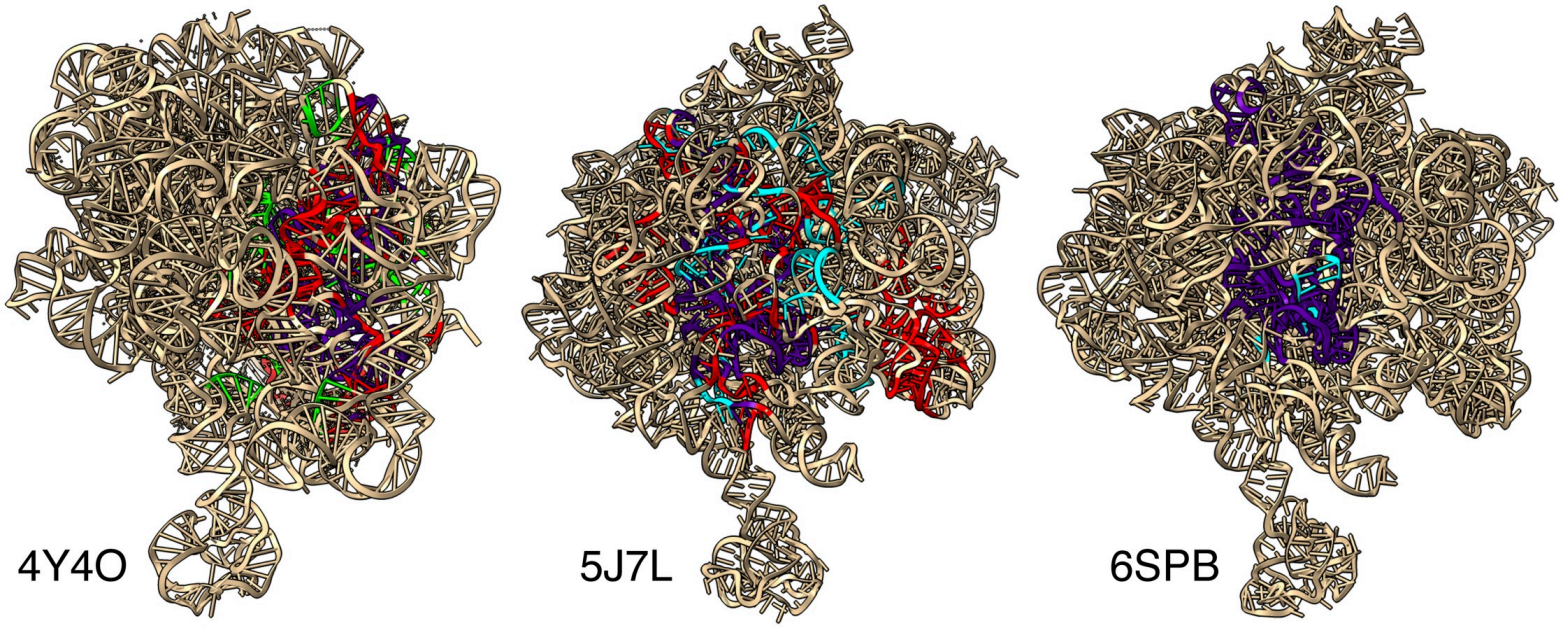
3 Largest ${\text{RIN}}_{\text{s}}^{\text{a}}$ in their contexts. The figure displays three 3D structures of ribosomal RNAs: 4Y4O (chain: 2A), 5J7L (chain: DA) and 6SPB (chain: A). The colored parts correspond to the 3 largest ${\text{RIN}}_{\text{s}}^{\text{a}}$ found in Dataset 3.137: RIN^a^_#1984_ in red, RIN^a^_#1983_ in cyan and RIN^a^_#1982_ in lime green. The overlap of two ${\text{RIN}}_{\text{s}}^{\text{a}}$ is colored in indigo. Additional information about those ${\text{RIN}}_{\text{s}}^{\text{a}}$ and their overlap is provided in Table 7.

Although a detailed study of those large ${\text{RIN}}_{\text{s}}^{\text{a}}$ falls out of the score of this paper, Fig 13 displays the 3 largest ${\text{RIN}}_{\text{s}}^{\text{a}}$ found in Dataset 3.137 in the contexts they have been found in, for illustrative purposes. Those 3 largest ${\text{RIN}}_{\text{s}}^{\text{a}}$ all have only 2 occurrences found in 3 different RNA chains. Table 7 provide additional information about those 3 largest ${\text{RIN}}_{\text{s}}^{\text{a}}$ and their respective overlaps (i.e. the largest common subgraph between each pair of RIN^a^).

**Table 7:** Additional information on the 3 largest ${\text{RIN}}_{\text{s}}^{\text{a}}$ found in Dataset 3.137. The colors correspond to ones used in Fig 13. The values for the overlaps correspond to the number of nodes shared between the ${\text{RIN}}_{\text{s}}^{\text{a}}$. The RNA chains are denoted using the name of the file (ex:4Y4O) plus the name of the chain (ex:2A).

				Overlap with RIN^a^:				
RIN^a^	Color	nodes	edges	#1984	#1983	#1982	Found in RNA chains:	
#1984	red	376	769	-	127	126	4Y4O,2A	5J7L,DA
#1983	cyan	236	491	127	-	227	6SPB,DA	5J7L,DA
#1982	green	228	473	126	227	-	6SPB,DA	4Y4O,2A

A more focused analysis of the biggest motifs shows how they are composed of interconnected A-minor motifs. In fact, all RINs with 100 nodes or more have an A-minor, with up to 8 for the largest one. This highlights the important role of A-minor geometric conformations to stabilize complex architectures associated with functional RNAs. It also suggests the existence of a selective pressure to conserve these structures and possibly the trace of convergent evolution.

### 3.5 Applications to RNA 3D module-based RNA structure prediction

As described earlier, we designed our method to be versatile by separating the rule system that define what structures should be extracted from the graph matching algorithms. We illustrated this versatility in section 3.3 with 3 RIN classes (RIN^abc^,RIN^ab^ and RIN^a^). In addition to RIN classes, we applied our method on another class of structures linked to the RNA 3D structure prediction problem: the *RNA 3D modules*.

*RNA 3D modules* are small RNA substructures involved in structural organization and ligand binding processes that can be leveraged in the prediction of a full 3D structure. The fragment-based method implemented by Parisien and Major in MC-Sym [33] constructs a full 3D structure from an augmented secondary structure by mapping the components of this secondary structure to a database of 3D structure fragments. The prediction of 3D modules has been shown to improve this class of methods by providing more informative fragments, namely in RNA-MoIP [7]. Further progress has since been made in this direction with recent improvements in *RNA 3D modules* identification in sequences [34, 35].

The main limitation of this type of method remains the difficulty of assembling a strong dataset of modules. RNA modules are typically identified by searching RNA 3D structures for recurrent subgraphs, a task to which CaRNAval should have been able to contribute. Unfortunately, as of now, no fragment-based method has been able to integrate long-range modules into a 3D structure prediction pipeline. Moreover, RNA modules do not need to include long range interactions, and many of the well characterized modules are entirely local, namely the kink-turn and g-bulged modules, and the published version of CaRNAval cannot be applied to the discovery of common subgraphs without long range interactions as its execution time would explode. However, the method presented in this paper does not have such limitation as demonstrated by the extraction of the RIN^a^ class.

**Fig 14:**
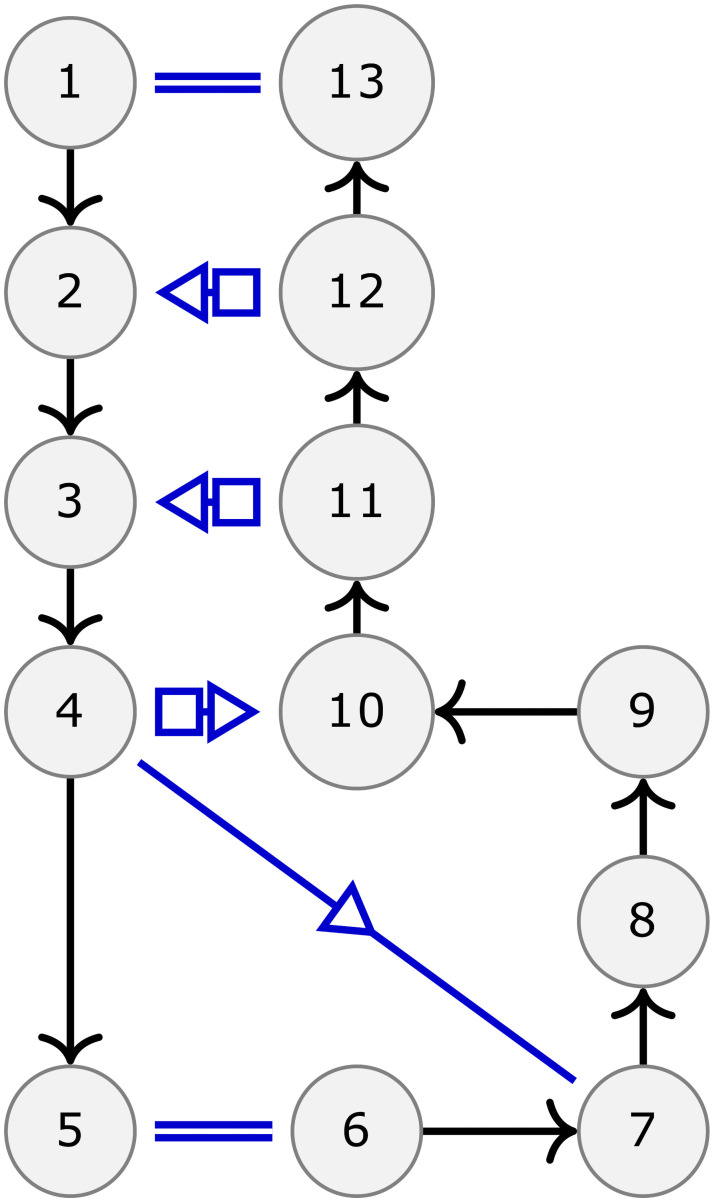
Kink-turn found in Dataset 3.137 with our method.

We adapted the set of rules of the RIN classes presented in section 3.2 to the problem, focusing on purely local *RNA 3D modules*, as a first approximation. The resulting set of rules would correspond, in our notation, to the RIN^xyb̄^ class, with b̄ being the constraint of having no long-range interactions. We relaxed rule z as it would have invalidated structures we are interested in such as the kink-turn that contains a bulge of backbone. Using this definition, we extracted 3387 structures with a total of 39513 occurrences from Dataset 3.137. Amongst those 3387 are structures we aimed at extracting such as the kink-turn displayed in Fig 14, which highlight the potential of our approach. However, those results also highlight a challenging aspect of *RNA 3D modules*. Indeed, several non-isomorphic structures can be labelled as a single *RNA 3D modules*. Please note that it is not specific to our approach: the RNA 3D Motif Atlas, the reference dataset for local modules, typically has multiple entries that match the definition of the kink-turn loop. As our method relies on exact graph matching to compare structures, an extra step of post processing needs to be added to the pipeline to process the structures found into a collection of *RNA 3D modules*. However, the design of this additional step is not trivial and falls out of the scope of this paper and so does the improvement of the set of rules used.

Even with the shortcomings we just mentioned, those results show that the modularity and the improved complexity allow for the tackling of this problem (cf. Table 5, for an indication of the size of the search space). Our method constitutes the first software able to discover both long-range and local RNA modules and as such, a significant step towards more accurate fragment-based prediction of 3D structure from sequence.

## 4 Conclusion

In this paper we present a novel method that can find arbitrarily large recurrent interaction networks (RINs) between two RNA structures, represented as graphs. Our graphical model encodes the base interactions found in the structure and the edges are labelled with a color representing the type of interaction according to the Leontis-Westhof classification [30]. We designed three novel graph matching algorithms (i.e. isomorphism, subgraph and maximal common subgraph algorithms) that leverage the information embedded in edge colors and apply these techniques to retrieve recurrent RNA base pairing networks (i.e. Recurrent Interaction Networks or RINs). Our methods improve by several orders of magnitude the computational efficiency compared to previous approaches. This technical breakthrough enables us to relax constraints used in previous studies to search for recurrent RNA motifs without pre-established assumptions.

To demonstrate the performance of our methods, we first successfully reproduce the results presented in CaRNAval and show that we can conduct the same analysis in a matter of hours instead of months. This achievement is an important milestone towards the release of a reliable online database of motifs for structural biologists studying the architecture and evolution of RNA structures. In particular, in light of the increasing number of new RNA 3D models deposited in the structural repositories (e.g. for the first time, more than 100 RNA-only structures have been released in the RCSB Protein Data Bank in 2020), our ability to quickly update our catalog of recurrent motifs (i.e. RIN) is key to maintaining this service up-to-date.

Then, we proceed to another computational experiment to highlight new opportunities offered by our technology. We take advantage of our improved computational efficiency to relax constraints previously set in earlier studies and expand the definition of a RIN. It enables us to search for larger classes of RINs like RIN^a^, which can span any number of SSEs and have any number of long range interaction (including none). By contrast, previous attempts could only search for RINs with exactly two SSEs and at least one long range interaction. This novel analysis allows us to revisit observations made in earlier studies. For instance, while the network of RINs found by CaRNAval had three clearly separated components, the new network computed using the generalized definition of ${\text{RIN}}_{\text{s}}^{\text{a}}$ is made of a single giant component connected to more than 95% of all recurrent structures. This information could be key to revealing the underlying architecture of the network of RINs and helping us identify evolutionary paths that would allow for the emergence of specific functional motifs.

Even though a complete rigorous analysis and contextualization of this data is unfortunately out of scope, we believe these observations provide enough support to justify further investigations. This data could be useful for evolutionary studies of ribosomes [23, 36–38], viroids structures [39] and the enhancement of motifs libraries for RNA design [8, 40]. As illustrated in sub-section 3.4.2, our algorithms could also contribute to identify higher-order RINs in ribosomal structures.

Yet, the development of additional theoretical models is warranted to assess the significance of the RINs detected. For instance, albeit our methods can efficiently extract recurrent motifs, it remains unclear if the redundancy stems from a selective pressure or from a composition bias in the input data set. To answer such question, we need to develop null models of graphical representations of RNA (sub-)structures (e.g., [41]) that itself deserves a full study on its own. Nonetheless, we designed our computational framework to accommodate such need in the future and maintained the independence of the definition of motifs to search for from the graph matching algorithms.

Finally, although our algorithms have been specifically developed for analyzing RNA base interaction networks, they could be customized to process other molecular structures (e.g. proteins) or more general biological networks (e.g. biological pathways). Therefore, we also hope that this contribution will inspire the development of other bioinformatics tools.

## Supporting information

S1 Text Algorithms for efficient graph matching of edge-colored graphs. Provides formal descriptions of all three graph matching algorithms presented in this papers with a complete proof of correctness for the maximal subgraph isomorphism algorithm with several explanatory diagrams. It also provides discussions on the complexity of all three algorithms and on the types of graphs they can be applied to.
(PDF)

S2 Text Extraction of Recurrent Structural Elements. Provides additional details about three auxiliary mechanisms of our method: the management of exceptions to the proper edge-coloring in data, the gathering of partial results and the parallelization of the pipeline.
(PDF)
