# Supplementary material for: Finding recurrent RNA structural networks with fast maximal common subgraphs of edge-colored graphs
Source: PLoS Comput Biol. 2021 May 28;17(5):e1008990. doi: 10.1371/journal.pcbi.1008990 (PMC8191989; doi:10.1371/journal.pcbi.1008990)
Supplement: S2 Text — Provides additional details about three auxiliary mechanisms of our method: the management of exceptions to the proper edge-coloring in data, the gathering of partial results and the parallelization of the pipeline. (PDF) [file pcbi.1008990.s002.pdf]

Finding recurrent RNA structural networks with fast maximal common subgraphs of edge-colored graphs

-S2 text-
Extraction of Recurrent Structural Elements

Antoine Soulé [1,2], Vladimir Reinharz [3],

Roman Sarrazin-Gendron [1], Jérôme Waldispühl [1,*]

and Alain Denise[4,5,*]

[1]School of Computer Science, McGill University, Montreal, Canada

[2]LiX, École Polytechnique, Paris, France

[3]Department of Computer Science, Université du Québec à Montréal, Montréal, Canada

[4]Laboratoire de recherche en informatique, Université Paris-Saclay - CNRS, Orsay, France

[5] Institute for Integrative Biology of the Cell (I2BC), Université Paris-Saclay - CEA - CNRS, Gif-sur-Yvette, France

∗ Corresponding Authors

April 2021

1 Management of exceptions to the proper edge-coloring

The annotation method of RNA structures obtained from biological experiments sometimes produces nodes involved in two base pairs of the same type. The labels of the edges of RNA 2D structure graphs containing such nodes do not form a *proper edge colouring* which is problematic for our graph-matching algorithms. Those violations might be artifacts from the interaction prediction method which is based on distance thresholds between the nucleobases' atoms. However, it is possible that those double interactions are actually biologically relevant and we thus propose a solution to handle them. From our observations, such node are very uncommon (a dozen nodes over the fifty-two thousands nodes of the complexes from the non-redundant RNA database maintained on RNA3DHub [1]). Moreover, in all the cases we observed, the nucleobases forming the same interaction twice was forming it with two other nucleobases consecutive in the backbone. As a consequence, we handle those exceptions by duplicating any connected component containing a problematic node as follows :

With $a, b, c \in N$ s.t. $\{a, b\}, \{a, c\} \in E_l$ and $b < c$, and κ the connected component containing them. We create $\kappa_a^!, \kappa_a^-, \kappa_a^+$ and κ_a^* with :

- $\kappa_a^! = \kappa - \{\{a, b\}, \{a, c\}\}$
- $\kappa_a^- = \kappa - \{\{a, c\}\}$
- $\kappa_a^+ = \kappa - \{\{a, b\}\}$
- $\kappa_a^* = \kappa \ with \ \{a, b\} \in E_{l^-}, \{a, c\} \in E_{l^+}$

Please note that $\{a, b\}$ and $\{a, c\}$ can always be compared by comparing the position of b and c in the backbone. κ_a^- is thus the version of κ where the "highest" edge of the two has been removed while κ_a^+ is thus the version of κ where the "lowest" edge of the two has been removed.

The labels of the edges of those four versions of κ form a *proper edge colouring* and represent three different interpretations of those exceptions :

- $\kappa_a^{!}$: both interactions are artifacts
- $\kappa_a^{-}, \kappa_a^{+}$: one interaction is valid, the other is an artifact
- κ_a^{*} : both interaction are valid, as a consequence those triangular structures are meaningful and thus should only be matched to similar structures

Those interpretations will impact how we handle several violations occurring in the same connected component. Let a and x two nodes that are incompatible with a *proper edge colouring* in the same connected component κ. $\kappa^{!}$ and κ^{*} will just stack leading to $\kappa_{ax}^{!}$ and κ_{ax}^{*}. However κ^{-} and κ^{+} need to cover every possibility leading to $\kappa_{ax}^{--}, \kappa_{ax}^{-+}, \kappa_{ax}^{+-}$ and κ_{ax}^{++}. This system is obviously extendable to any number of violation. Most observed violations are in separate connected components with only three of them being in the same.

This solution of duplicating some connected components does not rule out any possibility and thus offers the opportunity to compare the structures observed in each version. Moreover, the limited number of problematic nucleobases observed makes the cost negligible.

To avoid duplicating results, if the same structure is found in several versions of c, we only keep one occurrence. The conserved occurence is picked according to the following order: $\kappa^{!} > \kappa^{-} > \kappa^{+} > \kappa^{*}$. This order prioritizes the connected component our solution impacted the least so the fact that an occurence is mentioned has found in κ^{*} implies that the triangular structure was required. We also do not search common structure between different versions of the same component as they represent the same nucleotides of the same RNA.

Please note that we have been considering connected component in this section because f' may disconnect the RNA 2D structure graphs.

2 Gathering of partial results

The core of the process of transforming a set of maximal common subgraphs (mcsg) into a collection of recurrent interaction networks (RINs) relies on the application of the filtering function f_{RIN} to each mcsg in order to obtain the RINs inside it. We thus obtain a set of RINs from each mcsg found. We merge those sets of RINs so identical RINs (i.e. which canonical graphs are isomorphic) are merged into a single RIN combining all their occurrences (without duplicates). However, the *set of sets RINs found in the mcsgs* we obtain is not identical to the *set of RINs found in the dataset* we are seeking. To obtain the later, we have to correct two issues in the former.

A. First, the *set of sets RINs found in the mcsgs* may contain RINs with incomplete collections of occurrences. To put it differently, those RINs can be found in the dataset at positions that are not covered by any occurrences in their respective collections of occurrences. Those missing positions have been captured by the mcsgs but were "consumed" by/for another RIN. For instance, let us consider two graphs G and H and two RINs a and b such that:

1_A. the canonical graph of a is a subgraph of the canonical graph of b

2_A. b has two occurrences in H and one in G while a has two in both

Please note that because of 1_A, each occurrence of b induces an occurrence of a. Let us consider the occurrence of a in G that is not induced by an occurrence of b. This occurrence will be captured by at least two mcsgs, one for each of the occurrences of a in H. However the second occurrence of a in G may only be captured by mcsgs that are capturing the instances of b at the same time. As a consequence, the output of f_{RIN} when we applied it to those mcsgs will only contain b and not a. As a result, a might appear with only three occurrences instead of four.

However, this issue can easily be covered by checking the collections of occurrences of any such pairs of RINs a and b and creating any missing occurrence of a from the occurrences of b.

B. Second, the *set of sets RINs found in the mcsgs* may contain pairs of RINs a and b such that:

1_B. the canonical graph of a is a subgraph of the canonical graph of b

2_B. each occurrence of a is induced by an occurrence of b (to put it differently, a can only be found in the dataset inside occurrences of b)

In such case, we consider that a does not provide any additional information to the collection and thus that it needs to be removed for the sake of the readability of the collection of RINs.

The presence of such RINs in the *set of RINs found in the mcsgs* is due to the counterintuitive fact that a *maximal* common subgraph may contain a *"non-maximal"* RIN i.e. a RIN which canonical graph is a subgraph of another RIN that can be found at the same positions in the two graphs (this other RIN is actually found at those very positions but by another mcsg). This happens when the nodes of the pairs of nodes needed to capture the larger RIN have been used to form other pairs of nodes: the resulting common subgraph is indeed maximal but those other pairs of nodes happen to have been eliminated by f_{RIN}, thus losing maximality.

As a consequence, we need to filter the *set of RINs found in the mcsgs* to eliminate such RINs. This could be done by checking the dataset for pairs of RINs a and b that satisfy both conditions 1_B and 2_B and remove a but the fact that we completed the collections of occurrences to cover for issue A allows for a simpler solution. Indeed, once the collections of occurrences are completed, for all pairs of RINs a and b that satisfy condition 1_B, the number of occurrences of a cannot be less than the number of occurrences of b. Moreover, the number of occurrences of a first will be greater than the number of occurrences of b, except if the pair also satisfies condition 2_B. As a consequence, we can simply go through all pairs that satisfy 1_B, compare their number of occurrences and eliminate a if they are equal.

3 Parallel computing

The pipeline has been designed to support parallel computing as the problem is greatly compatible with it. Indeed, the production of each set of maximal common subgraphs of two RNA 2D structure graphs is independent and can be processed separately. Thus we divide the work between n cores between a *master* process et $n-1$ *workers*. The *workers* are provided with pairs of RNA 2D structure graphs by the *master* and send back the corresponding set of proto-RINs (i.e. they produce the corresponding set of maximal common subgraphs and then process it into the corresponding set of proto-RINs before sending it back). The *master* ensures that all pairs of RNA 2D structure graphs are processed and gather the results. Gathering the results includes the merging of partial results into the final collection. Rather than doing the merging in one go at the end of the computation which would let it with little to do when the *workers* are working, the *master* rather performs this task progressively as the *workers* send back their results.
All running times (i.e. original `CaRNAval`, RIN^{ab} and RIN^{a}) are measured on the same machine (Intel(R) Xeon(R) CPU E5-2667 0 @ 2.90GHz, Ubuntu 16.0.4 with 23 cores, total physical memory of 792 gigabytes). The numbers we provide are total consumptions (i.e. the sums of CPU time consumed for all the core).

Bibliography

[1] Anton Petrov. *RNA 3D Motifs: Identification, Clustering, and Analysis*. PhD thesis, Bowling Green State University, 2012.
